# Artificially sweetened beverages consumption and risk of obesity-related cancers: a wide-angled Mendelian randomization study

**DOI:** 10.3389/fnut.2024.1347724

**Published:** 2024-03-06

**Authors:** Xing Jin, Mengyue Wu, Shuangshuang Dong, Hui Liu, Haochuan Ma

**Affiliations:** ^1^Department of Laboratory Medicine, The Affiliated Hospital of Yangzhou University, Yangzhou University, Yangzhou, China; ^2^Medical College, Yangzhou University, Yangzhou, China; ^3^Department of Radiotherapy, General Hospital of Southern Theatre Command, Guangzhou, China; ^4^Department of Endocrinology, The Affiliated Hospital of Yangzhou University, Yangzhou University, Yangzhou, China; ^5^Department of Oncology, The Second Affiliated Hospital of Guangzhou University of Chinese Medicine, Guangdong Provincial Hospital of Traditional Chinese Medicine, Guangzhou, China; ^6^Guangdong Provincial Hospital of Chinese Medicine Postdoctoral Research Workstation, Guangzhou, China

**Keywords:** Mendelian randomization, artificially sweetened beverages, BMI, obesity, cancer risk, sugar

## Abstract

**Background:**

The impact of artificially sweetened beverages (ASBs) consumption on obesity-related cancers (ORCs) risk remains controversial. To address this challenging issue, this study employed wide-angle mendelian randomization (MR) analyses to explore the genetic causality between ASB consumption and the risk of ORCs, thereby effectively minimizing the impact of external confounders.

**Methods:**

We conducted a suite of analyses encompassing univariable, multivariable, and two-step MR to evaluate causal associations between ASB consumption (samples = 85,852) and risk of ORCs (total samples = 2,974,770) using summary statistics from genome-wide association studies (GWAS). Total, direct, and intermediary effects were derived by performing inverse-variance weighted (IVW), MR-Egger, weighted mode, weighted median, and lasso method. Additionally, we performed an extensive range of sensitivity analyses to counteract the potential effects of confounders, heterogeneity, and pleiotropy, enhancing the robustness and reliability of the findings.

**Results:**

Genetically predicted ASB consumption was positively associated with the risk of colorectal cancer (CRC, *p* = 0.011; OR: 6.879; 95% CI: 1.551, 30.512 by IVW) and breast cancer (*p* = 0.022; OR: 3.881; 95% CI: 2.023, 9.776 by IVW). Multivariable analysis yielded similar results. The results of the two-step MR unveiled that body mass index (BMI) assumes a pivotal role in mediating the association between ASB consumption and CRC risk (intermediary effect = 0.068, *p* = 0.024).

**Conclusion:**

No causal connection exists between ASB consumption and the majority of ORCs, in addition to CRC and breast cancer. Additionally, our findings suggest that BMI might be a potential mediator in the association between ASB consumption and CRC.

## Introduction

On July 14, 2023, a joint announcement from the World Health Organization (WHO), the International Agency for Research on Cancer (IARC), and the Joint Expert Committee on Food Additives (JECFA) classified aspartame as a Group 2B potential human carcinogen (1). This declaration raised significant concerns among both beverage manufacturers and consumers, especially since artificially sweetened beverages (ASB), notably those with aspartame, currently dominate a significant portion of the market (2, 3). Furthermore, for many individuals, ASB serves as the primary means of artificial sugar consumption, and epidemiological studies predominantly rely on the overall consumption of ASB to assess artificial sugar consumption (4).

The safety of ASB consumption has been a contentious topic for several decades. Numerous research endeavors have associated ASB consumption with a range of health conditions, including type 2 diabetes, cardiovascular diseases, neurodegenerative disorders, and obesity-associated cancers, with obesity-related cancers (ORCs) being particularly noteworthy (5–10). Obesity is a well-established predisposing factor for many malignancies, including liver, colorectal (CRC), ovarian, breast, esophageal, gastric, pancreatic, endometrial, kidney, and prostate cancers (11–19). Thus, they are also termed “ORCs”. Meanwhile, prior studies have shown a causal association between ASB consumption and obesity (20, 21). In this context, we hypothesized that consumption of ASB can alter weight, and ultimately result in cancer and that weight may be a mediating factor between the two. So far, there have been scattered studies exploring ASB consumption with ORCs (22–26). Nevertheless, some disadvantages exist. Firstly, most of the previous studies used dietary frequency questionnaires to obtain self-reported ASB intake information, which is subject to measurement error that could be attributed to recall and reporting bias (22). Furthermore, the majority of existing epidemiological research leans on case–control or cohort frameworks, intrinsically susceptible to biases (27, 28). Compounding the issue, there’s a lack of consensus in the findings across different studies. For instance, a French study linked higher ASB consumption to a slightly increased risk of ORCs, while an Australian study found no such association (4, 29). In light of these inconsistencies, Dr. Moez Sanaa, who heads the Food and Nutrition Standards and Scientific Advice Branch at WHO, underscores the necessity for more focused research and trials (1). This would pave the way for more definitive insights, aiding agencies, consumers, and producers in navigating this longstanding debate.

Mendelian randomization (MR), a commonly employed epidemiological approach, operates on the basic principle that alleles undergo random allocation during gamete formation (30). This approach leverages genotypes as instrumental variables (IVs) for ASB consumption to deduce causal relationships pertaining to tumorigenesis risk. Serving as a natural counterpart to a randomized controlled trial (RCT), it offers effect estimates that remain unadulterated by potential confounders (31). Consequently, we employed univariate, multivariate, and two-step MR to delve into the potential causal links between ASB consumption and ORCs, encompassing liver, thyroid, CRC, ovarian, breast (combined), estrogen receptor-positive (ER+) breast, ER- breast, esophageal, gastric, pancreatic, endometrial, kidney, and prostate cancers. As a complement to the current observational studies, we aim to provide a basis for defining public health policies related to ASB with a more accurate and comprehensive method.

## Methods

### Study design

**Figure 1:**
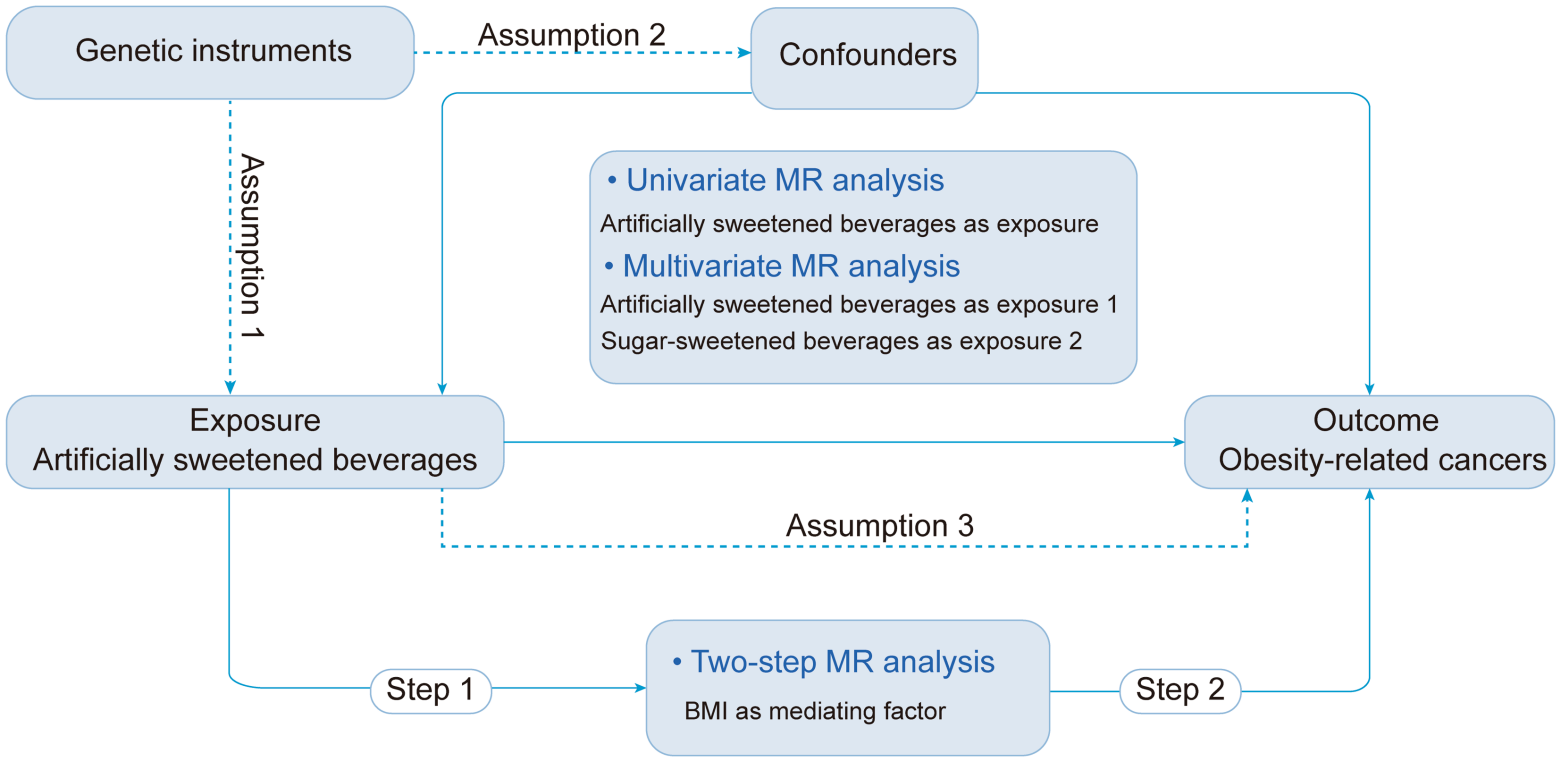
Study design overview and assumptions of the MR study. ASB, Artificially sweetened beverages; IVW, Inverse variance-weighted; MR, Mendelian randomization; SNPs, Single nucleotide polymorphisms.

The study design is shown in Figure 1. Utilizing aggregated data from the Genome-Wide Association Studies (GWAS), we initiated with a two-sample univariable MR analysis to investigate the potential association between ASB consumption and risk of ORCs, considering ASB consumption as the exposure and cancer incidence as outcome. Secondly, as mentioned in the introduction, we identified sugar-sweetened beverages (SSB) consumption as a potential confounding factor, and body mass index (BMI) as potential mediator to execute multivariate and two-step MR analyses, respectively (9, 32, 33). In the final phase, we integrated diverse sensitivity analyses to validate the dependability of the findings. The study was reported in accordance with the Guidelines for Strengthening the Reporting of Observational Studies in Epidemiology Using Mendelian Randomization (STROBE-MR) checklist (34).

### Study samples

Details regarding the data sources and sample sizes utilized in this study are succinctly outlined in Table 1. The study predominantly relied upon publicly accessible summary-level data, while ensuring that ethical approval was obtained for all original studies.

**Table 1:** Characteristics of exposure and outcome data.

Variables	Consortium	Year	Cases/controls	Population	Sex	Sample size
**Exposure**						
Artificially sweetened beverages (35)	UK Biobank	2019	Not relevant	European	Males and females	85,852
**Outcomes**						
Liver cancer (36)	UK Biobank	2021	165/456,111	European	Males and females	456,276
Thyroid cancer (37)	FinnGen	2023	1,783/287,137	European	Males and females	288,920
Colorectal cancer (38)	GECCO; CORECT; CCFR	2018	11,835/11,856	European	Males and females	23,691
Ovarian cancer (39)	OCAC	2017	25,509/40,941	European	Females	66,450
Prostate cancer (40)	PRACTICAL	2018	79,148/61,106	European	Males	140,254
Breast cancer (41)	BCAC	2015	46,785/42,892	European	Females	89,677
ER- breast cancer (42)	BCAC	2017	21,468/105,974	European	Females	127,442
ER+ breast cancer (42)	BCAC	2017	69,501/105,974	European	Females	175,475
Esophageal cancer (37)	FinnGen	2023	566/287,137	European	Males and females	287,703
Gastric cancer (37)	FinnGen	2023	1,307/287,137	European	Males and females	288,444
Pancreatic cancer (43)	Kaiser Permanente GERA; UK Biobank	2020	663/410,350	European	Males and females	411,013
Endometrial cancer (44)	ECAC; E2C2; UK Biobank	2018	12,906/108,979	European	Females	121,885
Kidney cancer (43)	Kaiser Permanente GERA; UK Biobank	2020	1,338/410,350	European	Males and females	411,688
Sugar-sweetened beverages (35)	UK Biobank	2019	Not relevant	European	Males and females	85,852
**Mediator**						
Body mass index (45)	UK Biobank; GIANT	2018	Unclear	European	Males and females	694,649

SNPs, Single-nucleotide polymorphisms; GECCO, Genetics and Epidemiology of Colorectal Cancer Consortium; CORECT, Colorectal Cancer Transdisciplinary Study; CCFR, Colon Cancer Family Registry; OCAC, Ovarian Cancer Association Consortium; PRACTICAL, Prostate Cancer Association Group to Investigate Cancer Associated Alterations in the Genome; BCAC, Breast Cancer Association Consortium; GERA, Genetic Epidemiology Research on Adult Health and Aging; GIANT, Genetic Investigation of ANthropometric Traits; ER, Estrogen receptor.

We sourced the GWAS summary statistics for ASB consumption from Zhong et al., with a sample size of 85,852 individuals of European ancestry from the UK Biobank (UKB), release 2019 (35). In this study, ASB consumption was obtained from a subset of participants in the UKB through the utilization of a 24-h recall questionnaire (Oxford WebQ) which encompassed the consumption of beverages containing non-nutritive sweeteners, such as aspartame, cyclamates, and saccharin.

To avoid sample overlap with the exposure (UKB), data for the majority of outcomes were collated from different consortia. The different cohorts were selected if the outcome data from UKB, further details can be found in Table 1 (36–44). Additionally, we incorporated GWAS datasets for potential confounding factor and mediator, namely SSB consumption and BMI, with the largest possible sample sizes. Details are stated as follows: BMI was extracted from a GWAS meta-analysis of between 221,863 and 806,810 largely unrelated adults of European ancestry from the Genetic Investigation of ANthropometric Traits (GIANT) consortium and UKB (45). In addition, data related to SSB consumption was also obtained from Zhong et al. (35). Furthermore, when the exposure variable data were incomplete, we removed subjects with undetectable values or imputed the undetectable values.

### Instrumental selection

For each analysis, we select eligible IVs for ASB by extracting the single nucleotide polymorphisms (SNPs) associated with each exposure at genome-wide significance (*p* < 5 × 10^−6^), with minor allele frequencies >1%, and not in linkage disequilibrium (LD) (*r*^2^ < 0.01). Furthermore, we filtered out SNPs that were associated with the results in the original GWAS (*p* < 5 × 10^−5^) to adhere to a crucial MR assumption: the impact of genetic variants on the risk of the outcome should be limited to risk factors and should not involve any alternative pathways. To ensure that the IVs were not associated with any potential confounding factors, we removed SNPs associated with confounders that might interfere the pathway between ASB and cancer. We considered five potential confounders, including type 2 diabetes mellitus, physical activity, smoking status, education level, and other dietary sources of sugar, all of which have been previously indicated to impact both ASB and cancer risk and widely used in relevant clinical and research settings (46–48). To filter out SNPs significantly linked with these confounders within the European ancestry, we utilized the PhenoScanner. The results of this filtration process were detailed in Supplementary Table S1. Finally, we calculated an F statistic to evaluate the overall effectiveness of the chosen SNPs in elucidating phenotypic variability, employing the equation: *F* = (beta/se)^2^. An *F* value exceeding 10 signifies that the instrumental SNPs possess considerable potency in mitigating potential biases, while an *F* value of 10 or below implies the SNPs might be weak IVs.

### Statistical analyses

Our statistical analysis was structured in three phases. Initially, we performed univariable MR analyses to assess the total effects. Next, we utilized multivariable and two-step MR analyses to differentiate the effects of the mediators on cancers, thereby determining the direct effects. In the final phase, we deployed a series of sensitivity analyses to gage the consistency of estimations and confirm the validity of the foundational MR assumptions. Additionally, the Steiger directionality test was implemented to ascertain the causal relationship between the exposure and outcome. For these analyses, we employed the R software packages: “TwoSampleMR”, “MVMR”, and “MendelianRandomization” (49).

#### Univariable MR

In detail, we used iteratively the inverse variance-weighted (IVW) method as the main analysis. However, it is important to note that the IVW results may be subject to bias if any of the SNPs exhibit horizontal pleiotropy. To address this concern and enhance the reliability of our findings, we employed three additional MR methods. One of these methods, MR-Egger, utilizes the slope coefficient of the Egger regression to estimate the causal effect, thereby offering a more robust estimate, even in the absence of any invalid instrumental variables. The weighted median method can provide protection against a notable proportion of invalid IVs, covering up to 50% of them. On the other hand, the application of the weighted mode method yields reliable estimations in cases where the relaxed assumption of IVs exhibits reduced bias and a diminished type I error rate. For situations where only a single genetic instrument is accessible, we resorted to the Wald ratio for the MR analysis (50).

#### Multivariable MR

Regarding the interplay of genetic instruments between consumption of ASB and SSB, we implemented multivariable MR analyses to discern the direct effects, which represent the influence on an outcome that can be specifically attributed to the exposure of interest rather than confounding factors (51). By combining the genetic instruments from the pertinent GWASs of both ASB and SSB, and subsequently clumping based on linkage disequilibrium (*R*^2^ < 0.01 within a window of 10,000 kb), we ensured the independence of the SNPs. Our primary method employed was the robust IVW approach, leveraging the difference of coefficients approach. In tandem, we assessed heterogeneity by using Q statistics.

#### Two-step MR

To evaluate the mediating impact of BMI, a two-step MR approach was employed (52). In the first step, genetic instruments for ASB consumption were used to deduce the causal impact of this exposure on BMI. In the second step, genetic instruments for BMI were used to determine the causal effect of the potential mediator on ORCs susceptibility. In instances where evidence suggested that ASB consumption influenced the mediator, which in turn influenced the cancer risk, we utilized the “product of coefficients” method to assess the indirect effect of ASB consumption on cancer risk via BMI. Standard errors for the indirect effects were derived by using the delta method.

### Sensitivity analysis

The results of the univariate, multivariate, and two-step MR analysis were confirmed following sensitivity analysis. We begin with Cochran’s Q test to assess the presence of heterogeneity. Subsequently, we used MR-PRESSO, accessible at https://github.com/rondolab/MR-PRESSO/, to identify the potential presence of horizontal pleiotropy, setting statistical significance thresholds at *p* values below 0.05. Further, an MR-Egger regression analysis was undertaken to explore the potential presence of directional pleiotropy bias. The Egger regression model’s intercept acts as a measure of the average pleiotropic impact across all genetic variants. An intercept not equaling zero (*p* < 0.05) is interpreted as evidence supporting the existence of pleiotropy. Analyses were done using R packages TwoSampleMR (version 0.4.10) and MRPRESSO (version 1.0).

## Results

### Univariable MR

**Figure 2:**
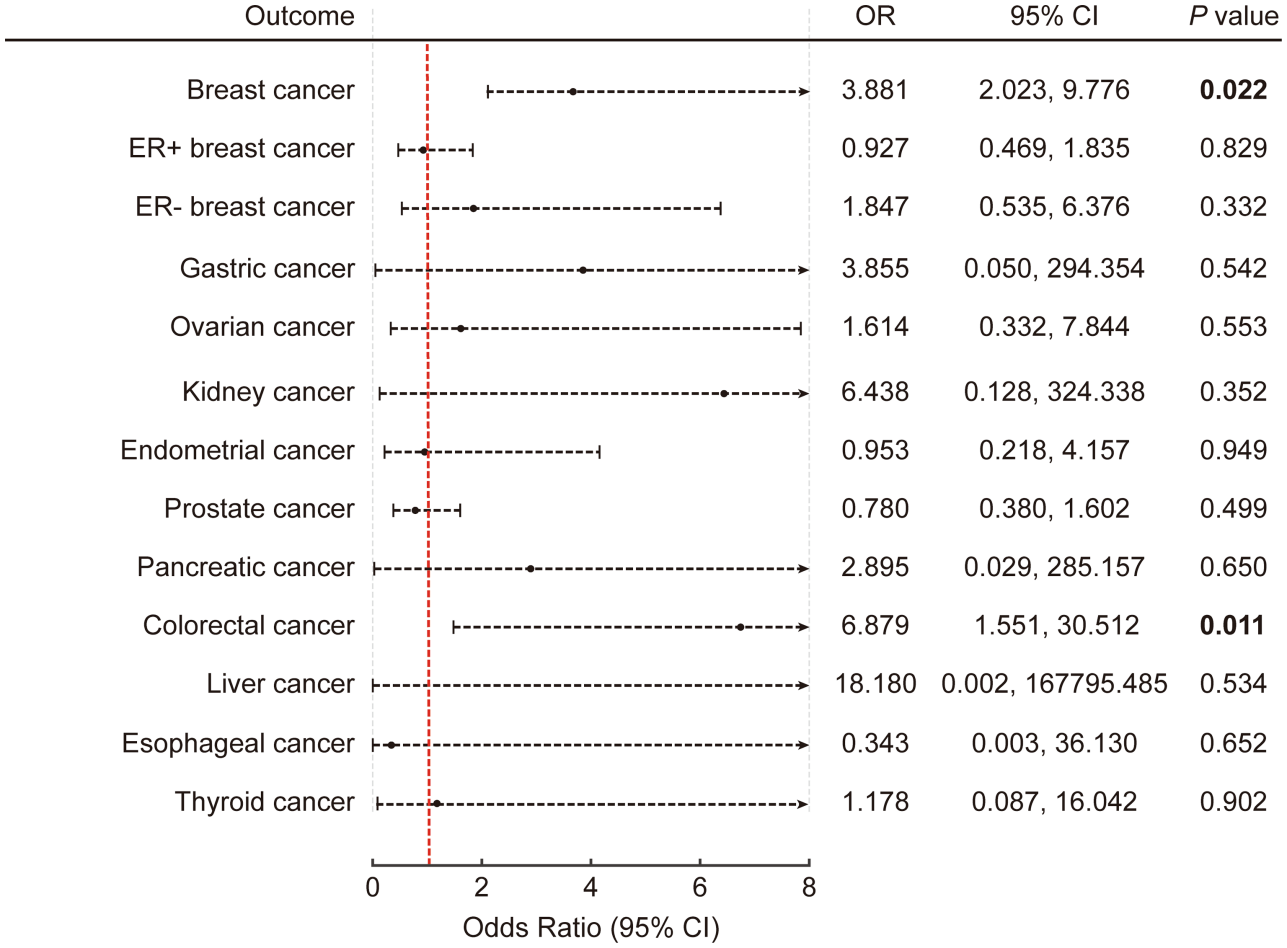
The causal association of artificially sweetened beverages with obesity-related cancers in univariable inverse variance-weighted model. The error bars represent 95% CI. All statistical tests were two-sided. *p* value < 0.05 was considered significant. CI, Confidence interval.

A list of all SNPs selected for inclusion or exclusion was provided in Supplementary Table S2 for replication. The *F* values were all greater than 20. In the context of univariable analysis, the primary findings were obtained using the radial IVW method, incorporating adjusted second-order weights in the final iteration. The findings were then presented as odds ratios (ORs) accompanied by 95% confidence interval (CI). Our analysis revealed that genetically predicted ASB consumption was positively associated with the risk of CRC (*p* = 0.011; OR: 6.879; 95% CI: 1.551, 30.512 by IVW) and breast cancer (*p* = 0.022; OR: 3.881; 95% CI: 2.023, 9.776 by IVW) (Figure 2; Supplementary Table S3). In addition, the rest of the results were negative.

### Multivariable MR

**Figure 3:**
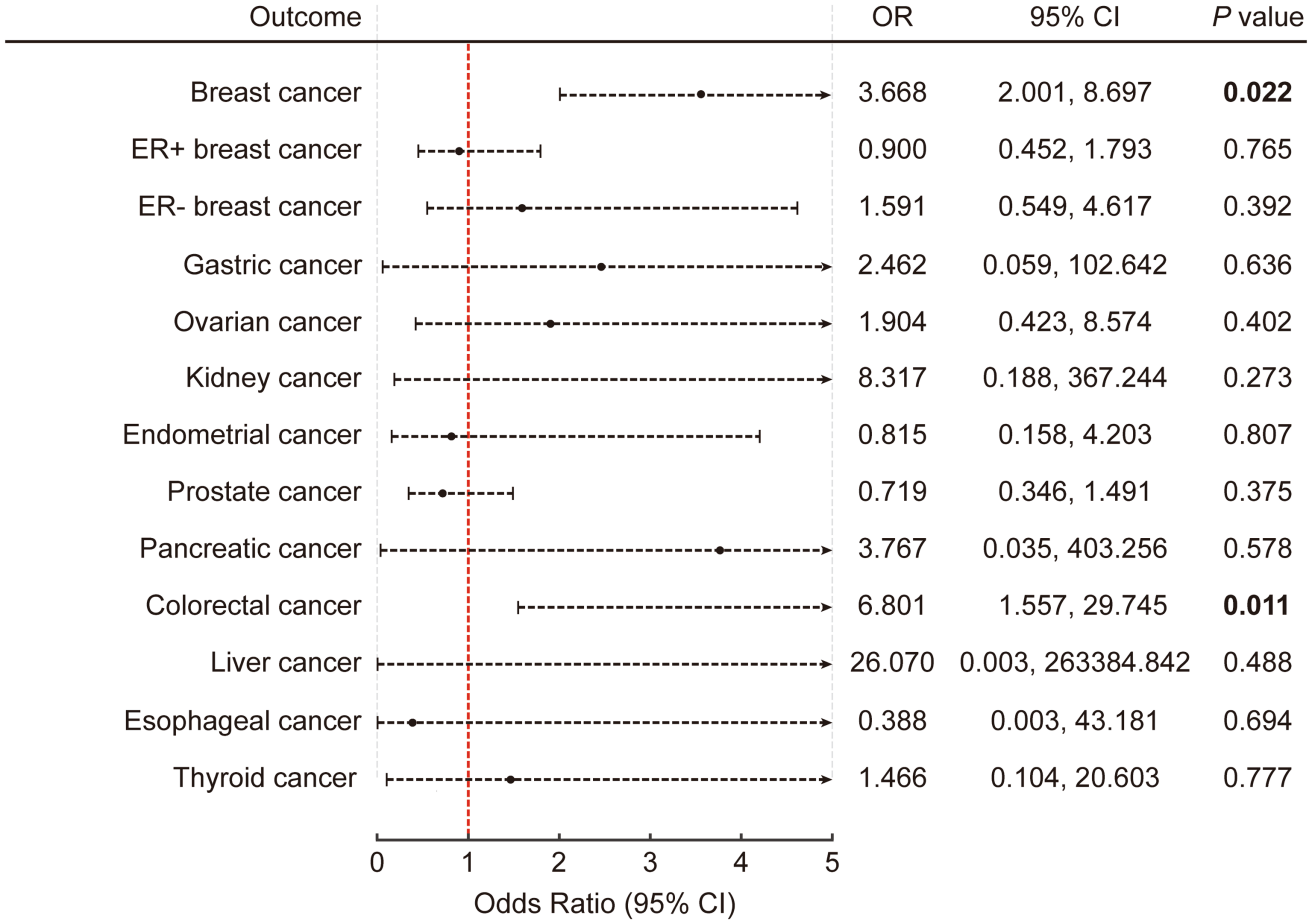
The causal association of artificially sweetened beverages with obesity-related cancers in multivariable inverse variance-weighted mode. The error bars represent 95% CI. All statistical tests were two-sided. *p* value < 0.05 was considered significant. CI, Confidence interval.

Supplementary Tables S2, S4 provide a comprehensive list of the independent instruments employed for multivariable MR. Similar to univariable MR, the main results in this section were derived from the multivariable robust IVW model with multiplicative random effects. The results identified a significant association between the consumption of ASB and the incidence of CRC (*p* = 0.011; OR: 6.801; 95% CI: 1.557, 29.745 by IVW) (Figure 3). This association was also observed specifically in breast cancer (*p* = 0.022; OR: 3.668; 95% CI: 2.001, 8.697 by IVW). In addition, the rest of the results were negative. All results are detailed in Supplementary Tables S5, S6.

### Two-step MR

**Figure 4:**
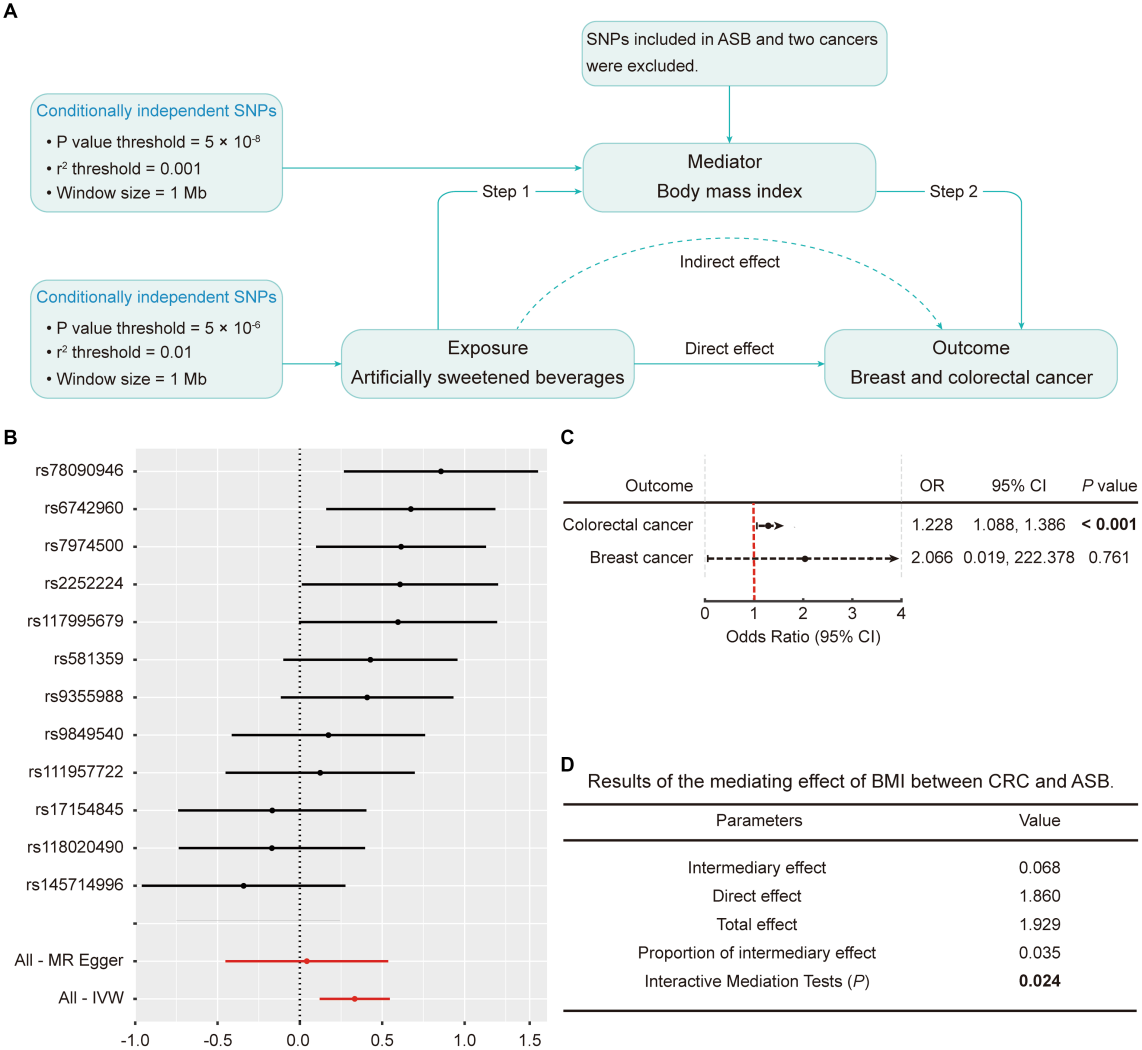
Mediation analysis of the effect of ASB on CRC and breast cancer via BMI. **(A)** Workflow of the two-step MR analysis. Step 1 estimated the causal effect of ASB on BMI, and step 2 assessed the causal effect of BMI on the risk of CRC and breast cancer. “Direct effect” indicates the effect of ASB on the risk of CRC and breast cancer after adjusting for the mediator. “Indirect effect” indicates the effect of ASB on the risk of CRC and breast cancer through the mediator. **(B)** Forest plot of individual and combined SNP MR-estimated effect sizes for the relationship between ASB and BMI. Summary MR estimates derived from the IVW, weighted median, and weighted mode for the effect of ASB on BMI are presented below the forest plot. **(C)** The effect of BMI on CRC and breast cancer risk derived from the IVW. **(D)** Results of the mediating effect of BMI between CRC and ASB. The error bars represent 95% CI. All statistical tests were two-sided. *p* value < 0.05 was considered significant. ASB, Artificially sweetened beverages; BMI, Body mass index; CI, Confidence interval; IVW, Inverse variance-weighted; SNPs, Single nucleotide polymorphisms.

To explore the potential mediating pathway from ASB consumption to cancer occurrence, we executed a two-step MR analysis, as illustrated in Figure 4A. In the first step, the results indicated a significant causal relationship between ASB consumption and BMI (β = 0.333; *p* = 0.002 by IVW) (Figure 4B; Supplementary Table S7). In the subsequent phase, we found causal evidence between BMI and CRC (*p* < 0.001; OR: 1.228; 95% CI: 1.088, 1.386 by IVW). Results are detailed in Figure 4C and Supplementary Table S8. Subsequently, we estimated the indirect effect of ASB consumption on CRC through BMI. We discerned that the mediation effect of BMI was evident in CRC (intermediary effect = 0.068, *p* = 0.024) (Figure 4D). In addition, there was no mediating effect of BMI between breast cancer and ASB consumption (Supplementary Table S9).

### Sensitivity analyses

For univariable, multivariable, and two-step MR, we performed sensitivity analyses to verify the results. Heterogeneity, as indicated by the Cochran’s Q statistic, was observed for ovarian cancer (*p* = 0.043 by IVW) in the univariable setting. Otherwise, no significant heterogeneity was found for all other estimates. The MR-PRESSO analysis did not pinpoint any potential outliers across the instrument effect. Moreover, the MR-Egger intercept analysis did not reveal any substantive evidence of directional pleiotropy. Collectively, across the three distinct MR frameworks, the robustness of our MR results was confirmed via the implementation of these sensitivity analyses. Comprehensive results can be found in Supplementary Tables S10–S14.

## Discussion

In this study, we have undertaken a wide-angled MR analysis, marking the most extensive and detailed examination to date, investigating the relationship between ASB consumption and the risk of ORCs. The study found no evidence of a causal relationship between ASB consumption and the majority of ORCs, however, ASB consumption can increase the risk of CRC and breast cancer. Additionally, through multivariate and two-step MR analyses, we evaluated the potential mediators. Our study pinpointed BMI as a pivotal mediator in the association between ASB consumption and CRC. Drawing from this, we propose that BMI potentially acts as a pathway through which ASB impacts the development of tumorigenesis.

In our study, no causal connection exists between ASB consumption and the majority of ORCs, which aligns with the conclusions of several prior clinical studies and meta-analyses. The NutriNet-Santé cohort study from France, which tracked 101,257 participants aged 18 and older for a median follow-up of 5.1 years using repeated 24-h dietary records, found no significant correlation between the consumption of ASB and ORCs risk after applying multiple-adjusted Fine and Gray risk models (24). A comprehensive systematic review and dose–response meta-analysis that incorporated 11 cohort studies resulted in 7 articles being chosen for the dose–response meta-analysis (33). This comprehensive assessment did not unearth any significant variance in ORCs risk with ASB consumption at a rate of 250 mL per day. Nevertheless, positive results were found with regard to CRC and breast cancer. A recent cohort study conducted by Debras et al. (4), which was published in PLOS Medicine, demonstrated a notable correlation between elevated consumption of ASB, particularly aspartame, and a 22% increased likelihood of developing breast cancer. Additionally, the study found a significant association between the consumption of ASB and the risk of ORCs, including colorectal, gastric, liver, esophageal, ovarian, and prostate cancers. This is partially consistent with our findings (CRC and breast cancer). We speculated that the following reasons might partly explain the outcomes. First, the association between ASB and the risk of CRC might be partly explained by its effect on overweight and obesity onset. Through two-step MR analysis, we have identified BMI as a crucial mediator in the association between ASB consumption and CRC. Recognizably, obesity is a pronounced risk factor for several cancers, notably those with obesity-related origins like esophageal, pancreatic, and colorectal cancers (20, 21). Concurrently, several studies have demonstrated a connection between artificial sweetener intake and weight gain, a relationship our research corroborates (53, 54). Yet, a more exhaustive examination is imperative to fully understand BMI’s mediating role, and the mechanism might be involved in tied to modifications in gut microbiota, the release of gut hormones, and metabolic aggregation (55, 56). For instance, research has shown that dietary patterns, including ASB, can impact the composition of gut microbiota, leading to the transformation of carcinogenic substances such as bile acids into metabolites like secondary bile acids and hydrogen sulfide, ultimately promoting the development of CRC (57). Moreover, the correlation between ASB and the risk of cancer cannot be solely elucidated by mechanisms associated with BMI, given that the majority of pertinent clinical investigations have adjusted the baseline BMI and weight fluctuations during follow-up (24, 58). In light of this, we conducted a thorough examination of various potential confounders, such as type 2 diabetes, physical activity, smoking habits, educational attainment, and sugar consumption from alternative dietary sources. Using PhenoScanner, we scrutinized the MR results both before and after these exclusions and found the conclusions consistent. This led us to consider that there may be other factors, such as the high glycaemic index or glycaemic load of ASB that could be influencing breast cancer risk instead of BMI. The glycaemic index has been linked to hyperinsulinaemia and type 2 diabetes, both of which have been implicated in the pathogenesis of breast cancer (59, 60). Consequently, we propose that the elevated risk of breast cancer associated with ASB may be attributed to dysregulated glucose metabolism and insulin dysfunction.

However, some studies presented conflicting results. An opposite conclusion was echoed in the MCC-Spain case–control study, which investigated a spectrum of cancers including colorectal, breast, prostate, stomach, and chronic lymphocytic leukemia (CLL) (61). Furthermore, findings from two prospective cohorts (the Nurses’ Health Study I and II) conducted in the United States revealed that the consumption of ASB did not exhibit a statistically significant association with an elevated risk of breast cancer (58). It’s worth noting that measurement error in dietary questionnaires is a non-negligible contributor to the risk of bias. While both studies (MCC-Spain and the Nurses’ Health Study) differentiated between SSB and ASB, the specific sweeteners and sugars in the assorted beverages were not distinctly categorized at the baseline. Moreover, as ASB, being sugar-free, is often perceived as a healthier alternative, individuals with specific conditions like diabetes might consume them more frequently. Given these nuances, it is imperative to exercise caution when interpreting these findings. Moreover, it should be noted that the connection between ASB and other types of tumors remains an open question. On the one hand, the presence of both intra- and inter-tumor heterogeneity, particularly in ovarian cancer, contribute to the differences in outcomes (62). On the other hand, additional research is required to elucidate the mechanisms through which ASB impacts various types of tumors. In addition to body mass index and high glycaemic index, mechanisms involving inflammation, angiogenesis, promotion of DNA damage, and inhibition of apoptosis may also play a significant role in this phenomenon (63, 64).

Our study stands out as the first attempt at leveraging MR analysis to explore the causal link between ASB consumption and ORCs risk, as far as our current understanding goes. The inherent strength of MR analysis lies in its diminished susceptibility to reverse causation and confounding, particularly when compared with observational studies. This ensures more rigorous evidence for causation, making it an indispensable tool for shaping public health policies. Furthermore, our design employed a wide-angled analysis by incorporating a plethora of MR methods and extensive sensitivity analyses. Such multi-faceted approaches are pivotal for addressing heterogeneity, confounding factors and pleiotropy, thereby bolstering the robustness and dependability of our results. We also harnessed MVMR and two-step MR techniques to probe into potential mediators, offering a more intricate understanding of the underlying mechanism. Demographic stratification bias is improbable to have influenced our findings, as the GWAS predominantly enlisted participants of European descent, and the demographic structure was accounted for through genetic principal components adjustment.

While our study provides significant insights, certain limitations warrant consideration. Firstly, the data for our study, sourced from the publicly available GWAS database, could potentially constrain our capacity to delve into the effects of dose stratification on the results. Given that ASB consumption might significantly impact tumor development risk, there’s a pressing need for an expanded body of clinical research that zeroes in on the dose–response gradient of ASB intake and its potential repercussions on cancer susceptibility. Moreover, pleiotropy poses a potential concern in any MR study. Nevertheless, we have adopted multiple strategies to guarantee the robustness of our findings. These strategies include leveraging MR-PRESSO to filter out outliers and undertaking sensitivity analyses using tools such as Cochran’s Q statistic, weighted median method, and MR-Egger methods. Lastly, as already mentioned, the study cohorts incorporated in our research exclusively comprised individuals of European descent. This demographic focus may limit the generalizability of our findings to broader racial and ethnic groups.

## Conclusion

In conclusion, no causal connection exists between ASB consumption and the majority of ORCs, in addition to CRC and breast cancer. Additionally, our findings suggest that BMI might be a potential mediator in the association between ASB consumption and CRC. Compared to prior observational studies, our research provides more robust evidence with fewer confounding factors. These insights will be instrumental in enriching the knowledge base of relevant entities, manufacturers, and consumers about the potential carcinogenicity of ASB.

## Data availability statement

The original contributions presented in the study are included in the article/Supplementary material, further inquiries can be directed to the corresponding authors.

## Ethics statement

Ethical approval was not required for the studies involving humans because all data used in the study are from public databases, there was no need for a separate ethical approval for this study. The studies were conducted in accordance with the local legislation and institutional requirements. Written informed consent for participation was not required from the participants or the participants’ legal guardians/next of kin in accordance with the national legislation and institutional requirements because all data used in the study are from public databases, there was no need for a separate informed consent for this study.

## Author contributions

XJ: Data curation, Formal analysis, Writing – original draft. MW: Data curation, Formal analysis, Validation, Writing – original draft. SD: Data curation, Software, Visualization, Writing – original draft. HL: Conceptualization, Methodology, Supervision, Writing – review & editing. HM: Funding acquisition, Methodology, Project administration, Supervision, Writing – review & editing.
