# Supplementary figures and images for: Artificially sweetened beverages consumption and risk of obesity-related cancers: a wide-angled Mendelian randomization study
Source: Front Nutr. 2024 Mar 6;11:1347724. doi: 10.3389/fnut.2024.1347724 (PMC10959093; doi:10.3389/fnut.2024.1347724)

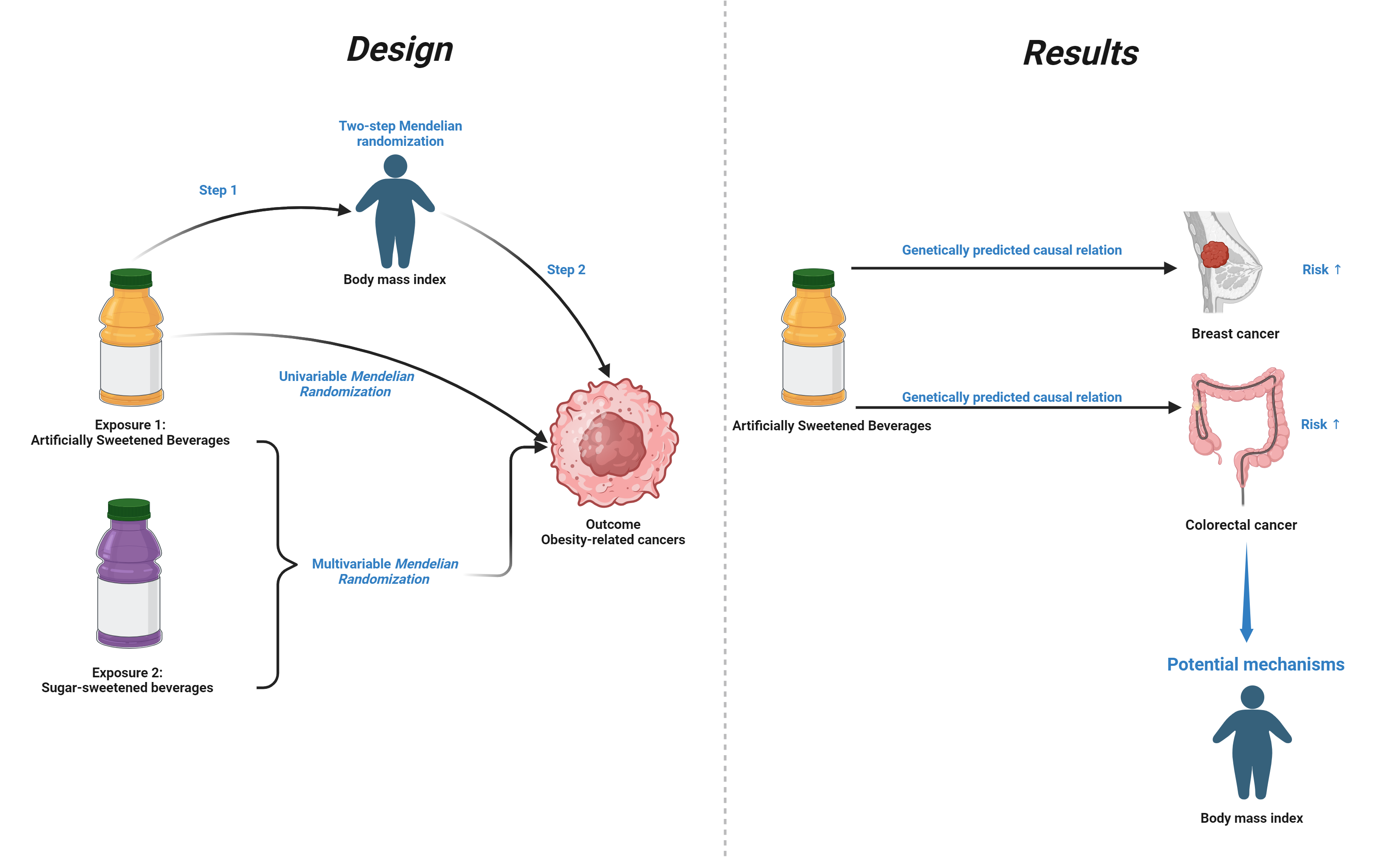

Supplement: Supplementary file 2 [file Image_1.PNG]
